# Chemosaturation with percutaneous hepatic perfusion is effective in patients with ocular melanoma and cholangiocarcinoma

**DOI:** 10.1007/s00432-020-03289-5

**Published:** 2020-06-20

**Authors:** Leon Schönfeld, Jan B. Hinrichs, Steffen Marquardt, Torsten Voigtländer, Cornelia Dewald, Wolfgang Koppert, Michael P. Manns, Frank Wacker, Arndt Vogel, Martha M. Kirstein

**Affiliations:** 1 Department of Gastroenterology, Hepatology and Endocrinology, Hannover Medical School, Carl-Neuberg-Str. 1, 30625 Hannover, Germany; 2 Institute for Diagnostic and Interventional Radiology, Hannover Medical School, Hannover, Germany; 3 Clinic for Diagnostic and Interventional Radiology, Henriettenstift, Hannover, Germany; 4 Department of Anesthesiology, Hannover Medical School, Hannover, Germany; 5 1st Department of Medicine, University Medical Center Schleswig-Holstein, Lübeck, Germany

**Keywords:** Percutaneous hepatic perfusion, Cholangiocarcinoma, Ocular melanoma

## Abstract

**Background:**

Chemosaturation with percutaneous hepatic perfusion (CS-PHP; Hepatic CHEMOSAT® Delivery System; Delcath Systems Inc, USA) is a novel interventional procedure, which delivers high doses of melphalan directly to the liver in patients with liver tumors while limiting systemic toxicity through hemofiltration of the hepatic venous blood. We have previously shown promising efficacy for patients with ocular melanoma (OM) and cholangiocarcinoma (CCA) within our single-center and multi-center experiences. The aim of this study was to analyze the safety and efficacy of CS-PHP after 141 treatments at Hannover Medical School, Germany.

**Methods:**

Overall response rates (ORR) were assessed according to Response Evaluation Criteria In Solid Tumors (RECIST1.1). Median Overall survival (mOS), median progression-free survival (mPFS), and median hepatic PFS (mhPFS) were analyzed using the Kaplan–Meier estimation.

**Results:**

Overall, 60 patients were treated with CS-PHP in the salvage setting from October 2014 until January 2019 at Hannover Medical School with a total of 141 procedures. Half of the patients were patients with hepatic metastases of ocular melanoma (OM) (*n* = 30), 14 patients had CCA (23.3%), 6 patients had hepatocellular carcinoma (10%), and 10 patients were treated for other secondary liver malignancies (16.7%). In total, ORR and disease stabilization rate were 33.3% and 70.3% (*n* = 25), respectively. ORR was highest for patients with OM (42.3%), followed by patients with CCA (30.8%). Independent response-associated factors were normal levels of lactate dehydrogenase (odds ratio (OR) 13.7; *p* = 0.015) and diagnosis with OM (OR 9.3; *p* = 0.028). Overall, mOS was 9 months, mPFS was 4 months, and mhPFS was 5 months. Patients with OM had the longest mOS, mPFS, and mhPFS with 12, 6, and 6 months, respectively. Adverse events included most frequently significant, but transient, hematologic toxicities (80% of grade 3/4 thrombopenia), less frequently hepatic injury up to liver failure (3.3%) and cardiovascular events including two cases of ischemic insults (5%).

**Conclusion:**

Salvage treatment with CS-PHP is safe and effective particularly in patients OM and CCA. Careful attention should be paid to possible, serious hepatic, and cardiovascular complications.

**Electronic supplementary material:**

The online version of this article (10.1007/s00432-020-03289-5) contains supplementary material, which is available to authorized users.

## Introduction

The Hepatic CHEMOSAT® Delivery System (Delcath Systems Inc., New York, NY, USA) is an innovative medical device for the treatment of patients with unresectable primary or secondary liver tumors. The system is used to perform chemosaturation percutaneous hepatic perfusion (CS-PHP), in which a high dose of the chemotherapeutic agent melphalan is delivered directly to the liver while limiting systemic exposure.

The efficacy of CS-PHP has been demonstrated in a randomized phase III trial in patients with hepatic metastases of ocular melanoma (OM) and cutaneous melanoma. CS-PHP significantly prolonged median progression-free survival (mPFS), median hepatic progression-free survival (mhPFS), and response rate compared to best alternative care (BAC) (Hughes et al. 2016). Within a phase I study including patients with solid tumors, amongst others hepatobiliary cancer, treatment with CS-PHP resulted in an overall response rate (ORR) of 30% including two complete responses (Pingpank et al. 2005). Since 2012, an improved second-generation filter is available with increased filtration efficacy. First results from prospective, randomized-controlled trials including the second-generation filter showed an acceptable safety and toxicity profile in 35 patients treated for liver metastases from ocular melanoma (Meijer et al. 2019).

In the real-life setting, several retrospective studies have investigated the safety and efficacy of CS-PHP among various secondary and primary liver tumors with promising results (Artzner et al. 2019; Kirstein et al. 2017; Vogl et al. 2014). In patients with OM, mOS and median progression-free survival (mPFS) reached up to 27 and 11 months, respectively (Artzner et al. 2019). Within our previous single-center experience, we have shown a good efficacy for patients with OM, but for patients with biliary tract cancer as well (Kirstein et al. 2017). In a subsequent multi-center study, we have exclusively investigated 15 patients with cholangiocarcinoma (CCA) treated with CS-PHP in a salvage setting and achieved a considerable ORR of 20% and a median OS of 8 months (Marquardt et al. 2019).

The aim of this study was to analyze the safety and efficacy of CS-PHP after 141 treatments in patients with primary and secondary hepatic tumors as last-line treatment at Hannover Medical School, Germany.

## Patients and methods

### Patient selection and data acquisition

In all patients, CS-PHP was regarded as the most appropriate therapy for the salvage setting after discussion within a multidisciplinary local tumor board. Criteria for a CS-PHP were adequate hematologic, renal, and hepatic function (hemoglobin > 8 g/dL; leukocyte count > 2 thsd/μL; platelets > 50 thsd/μL, serum creatinine > 60 µmol/L, bilirubin ≤ 3 × upper limit of normal [ULN], maximum Child–Pugh A). Patients were not treated, if they had a history of transient ischemic attacks, heart failure with a left-ventricular ejection fraction < 40%, or significant chronic obstructive or restrictive pulmonary disorder.

Patient data were retrospectively evaluated for baseline characteristics and therapies using clinical, imaging, and laboratory reports. Toxicity was assessed according to the Common Terminology Criteria for adverse Events (CTCAEv5.0) (https://ctep.cancer.gov/protocoldevelopment/electronic_applications/docs/CTCAE_v5_Quick_Reference_5x7.pdf). Radiological response was assessed according to Response Evaluation Criteria In Solid Tumors 1.1 (RECIST1.1) and additionally according to modified Response Evaluation Criteria In Solid Tumors (mRECIST) for HCC (Eisenhauer et al. 2009; Lencioni and Llovet 2010). Disease stabilization was defined as stable disease or response according to RECIST1.1. Overall survival (OS) was analyzed from first diagnosis and first CS-PHP until last follow-up or death. Progression-free survival (PFS) was analyzed from first CS-PHP until first radiological progression according to RECIST1.1 until last follow-up or death, whichever occurred first. Hepatic PFS (hPFS) was analyzed from first CS-PHP until first radiological hepatic progression, last follow-up, or death, whichever occurred first. Information about deaths were obtained from registration offices. Data cut-off was the 1st May 2019. The study protocol conformed to the ethical guidelines of the 1975 Declaration of Helsinki as reflected in a priori approval by the appropriate institutional review committee. Part of this study population (*n* = 29; 54 interventions) has previously been included and described within our single-center experience (Kirstein et al. 2017).

### Procedures

Imaging—either computed tomography (CT) or magnetic resonance imaging (MRI) with slice thickness from 1 to 5 mm—was performed on an average of eight weeks after CS-PHP. Contraindications for re-treatment were progressive disease (PD) as assessed by RECIST1.1 or intolerance to the procedure. The Hepatic CHEMOSAT® Delivery System was used to conduct CS-PHP in general anesthesia in an interventional angiography suite. The procedure was performed according to the company`s recommendations (Brochure Chemosaturation, Delcath Systems Inc., New York, NY, USA). Patients received single-shot antibiotics peri-interventionally and granulocyte-colony-stimulating factor (G-CSF) 24–72 h post-intervention due to the high rates of neutropenia.

### Statistical analysis

Statistical analyses were performed using SPSS 26.0 (SPSS Inc., Chicago, IL, USA). Continuous data were represented as median and interquartile range (IQR). Continuous, related data were tested for differences using the Wilcoxon test. Differences between categorical variables were calculated using Pearson’s Chi-square test. Binary logistic regression was performed in the forward selection method to predict response-associated factors including categorical variables and continuous variables, which were dichotomized by median or lower limit of normal into dummy variables. Survival was assessed using the Kaplan–Meier estimation. Comparison was made using the log-rank (Mantel–Cox) test. A probability (*p*) value less than 0.05 was considered significant.

## Results

### Patient characteristics

Overall, 60 patients were treated with CS-PHP from October 2014 to January 2019 at Hannover Medical School with a median follow-up of 27 months. Median age at first CS-PHP was 60.5 (IQR 52–66) years. Half of the patients had metastases of OM (*n* = 30), followed by patients with CCA (*n* = 14; 23.3%) and HCC (*n* = 6; 10%). Patients treated with CS-PHP for other secondary malignancies included colorectal cancer (*n* = 2; 3.3%), pancreatic cancer (*n* = 2; 3.3%), periampular carcinoma (*n* = 2; 3.3%), neuroendocrine tumors (*n* = 2; 3.3%), and breast and endometrial cancer (each *n* = 1; 1.7%). Seven patients had extra-hepatic tumor manifestations (11.9%) including bone (*n* = 4), pulmonary (*n* = 2), and cutaneous (*n* = 1) metastases. Patients with hepatic metastases of OM had significantly higher levels of lactate dehydrogenase (LDH) (*p* = 0.013), whereas other demographics were similar between the tumor entities (Table 1).

**Table 1:** Demographics

	Total		OM		CCA		HCC		Others		
	*n*	%	*n*	%	*n*	%	*n*	%	*n*	%	*p*
Total	60										
Female	36	60.0	21	70.0	6	42.9	1	16.7	4	40.0	
Male	24	40.0	9	30.0	8	57.1	5	83.3	6	60.0	0.112
LDH											
≤ 247 U/L	20	33.9	5	17.2	6	42.9	5	83.3	4	40.0	
> 247 U/L	39	66.1	24	82.8	8	57.1	1	16.7	6	60.0	0.013
Tumor volume											
> 30%	12	20.3	8	27.6	2	14.3	1	16.7	1	10.0	
≤ 30%	47	79.7	21	72.4	12	85.7	5	83.3	9	90.0	0.6579
Extra-hepatic spread											
No	52	88.1	24	82.8	13	92.9	6	100.0	9	90.0	
Yes	7	11.9	5	17.2	1	7.1	0	0	1	10.0	0.585
ECOG PS											
0	41	69.5	19	65.5	12	85.7	4	66.7	6	60.0	
1–2	18	30.5	10	34.5	2	14.3	2	33.3	4	40.0	0.493
Number of procedures											
1–2	38	63.3	16	53.3	9	64.3	5	83.3	8	80.0	
> 2	22	36.7	14	46.7	5	35.7	1	16.7	2	20.0	0.317

In total, 141 CS-PHPs were performed with a maximum of seven procedures in one patient. Most patients were treated with at least two procedures (*n* = 118; 83.7%). Median time between first procedure and second procedure was 63 (IQR 45–98) days, and median time between first procedure and first imaging control was 50 (IQR 38–75) days. Median time between first diagnosis and first CS-PHP was 25 (IQR 9–61.75) months and all patients had been extensively pre-treated with standard therapies, indicating that CS-PHP was performed in a salvage setting following the use of standard therapies (supplemental Table 1).

### Response assessment

Overall, 54 patients (90%) were available for radiological response assessment. One patient with OM died due to sepsis shortly after the first CS-PHP. Two other patients with OM died due to rapid tumor progression. Both patients had a high tumor burden as assessed by LDH (3370 U/L and 3280 U/L) and tumor volume (72.9% and 32.1%). The remaining three patients were lost to follow-up before imaging could be performed.

**Fig. 1:**
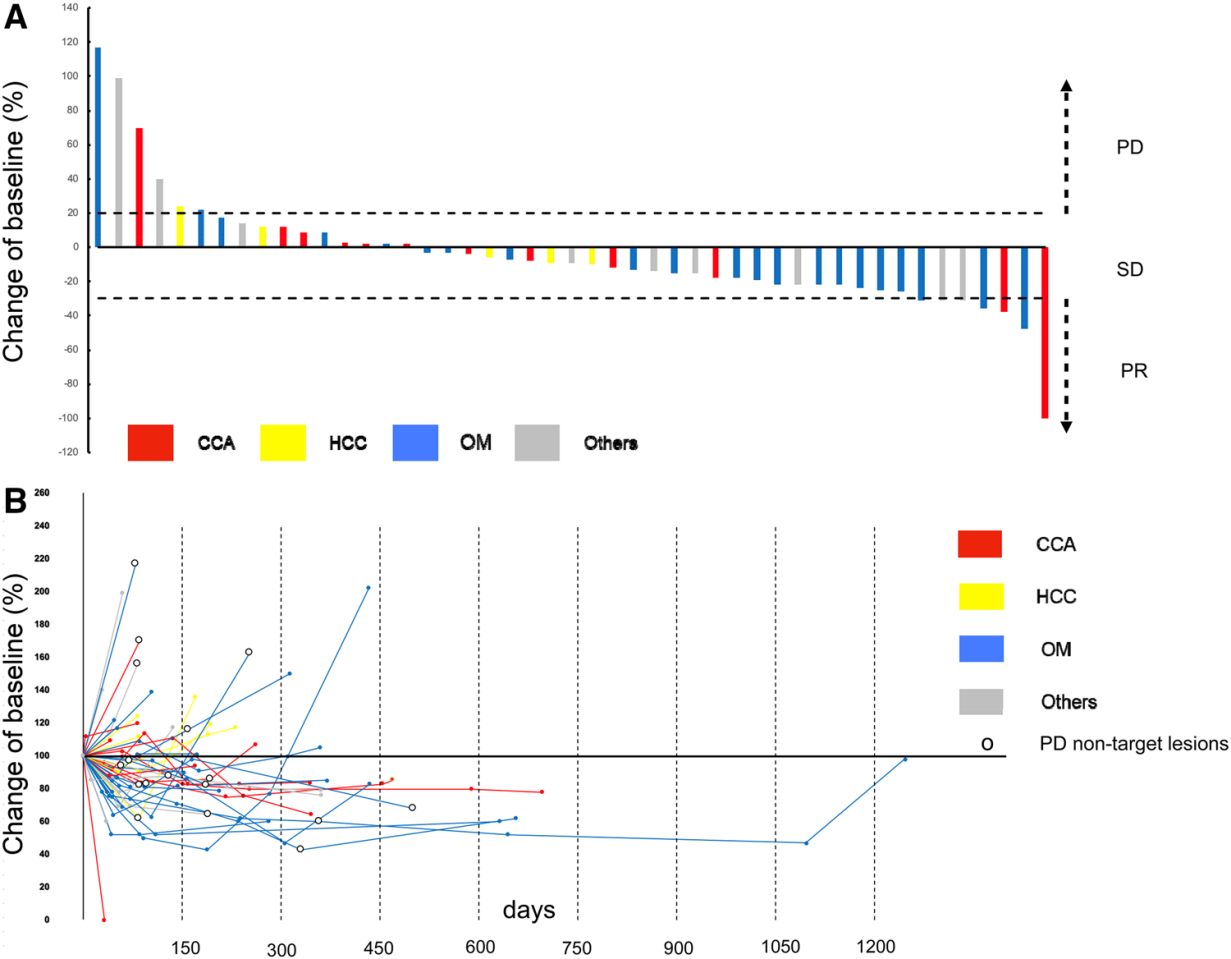
Response assessment. **a** Waterfall plot of baseline target lesions after first CS-PHP (maximum percentage change in baseline target lesions) with an overall response assessment including non-target lesions as indicated above the bars. Dashed lines are thresholds for progressive disease (PD) and partial response (PR). SD = stable disease. **b** Spider plot showing changes from baseline target lesions. White circles indicate PD of non-target lesions

After the first CS-PHP, 14 patients had a response (25.9%), 25 patients (46.3%) had stable disease, and 15 patients (*n* = 27.8%) had progressive disease (Fig. 1a). Among the patients with PD, 12 patients had only hepatic PD, whereas seven patients had extra-hepatic and/ or hepatic PD after the first CS-PHP.

Overall, disease stabilization was achieved in 38 patients (70.3%). The overall response rate (ORR) was 33.3% (*n* = 18) in total. The ORR was in trend higher for patients with OM (*n* = 11; 42.3%) compared to the other patients (*n* = 7; 25.0%; *p* = 0.178). The ORR of patients with CCA was 30.8% (*n* = 4) and 33.3% for patients with other secondary malignancies than OM (*n* = 3), whereas no patient with HCC had a radiological response according to RECIST1.1 and mRECIST. One patient with CCA had a complete response following the first CS-PHP. Unfortunately, this patient was lost to follow-up in the long-term course. Percentage changes of target and of non-target lesions are represented in Fig. 1b.

Using binary regression including variables reflecting performance status (ECOG) and age, tumor burden (tumor volume, LDH, c-reactive protein levels), tumor entity (OM vs. non-OM), and toxicity, normal levels of LDH (odds ratio (OR) 13.66; *p* = 0.015) and diagnosis with OM (OR 9.27; *p* = 0.028) were identified as predictors of achieving a response. In contrast, age ≥ 65 years at first CS-PHP was in trend negatively associated with response (OR 0.221; *p* = 0.063) (Table 2). Levels of LDH correlated with tumor burden in patients with OM: all patients with normal levels of LDH had a tumor volume ≤ 30%, whereas 76.2% (*n* = 16) of the patients with elevated levels of LDH had a tumor volume > 30%.

**Table 2:** Predictors of achieving a radiological response

Factors	OR	*p*	95% CI
ECOG 0 vs. 1–2	0.359	0.209	0.078–1.772
OM vs. non-OM	9.265	0.028	1.265–67.862
LDH normal vs. nncreased	13.658	0.015	1.652–112.862
Tumor volume > 30% vs. ≤ 30%	1.527	0.669	0.220–10.612
Age at first CS-PHP ≥ 60.5 vs. < 60.5 years	0.221	0.063	0.045–1.087
Grade 3/4 thrombopenia following 1^st^ CS-PHP	1.727	0.515	0.334–8.933
Grade 3/4 leucopenia following 1st CS-PHP	1.149	0.891	0.157–8.434
CRP normal vs. increased	3.75	0.12	0.708–19.867

OR = Odd`s ratio

### Survival

In total, mOS was 56 months from first diagnosis and 9 months from first CS-PHP (*n* = 60). mPFS was overall 4 months (135 days), whereas mhPFS was 5 months (169 days) (*n* = 55). Patients treated for hepatic metastases of OM had numerically longer mOS (12 vs. 8 months; *p* = 0.893), longer mPFS (6 vs. 3 months; 0.539), and a longer mhPFS (6 vs. 5 months; *p* = 0.657) compared to the other patients.

**Fig. 2:**
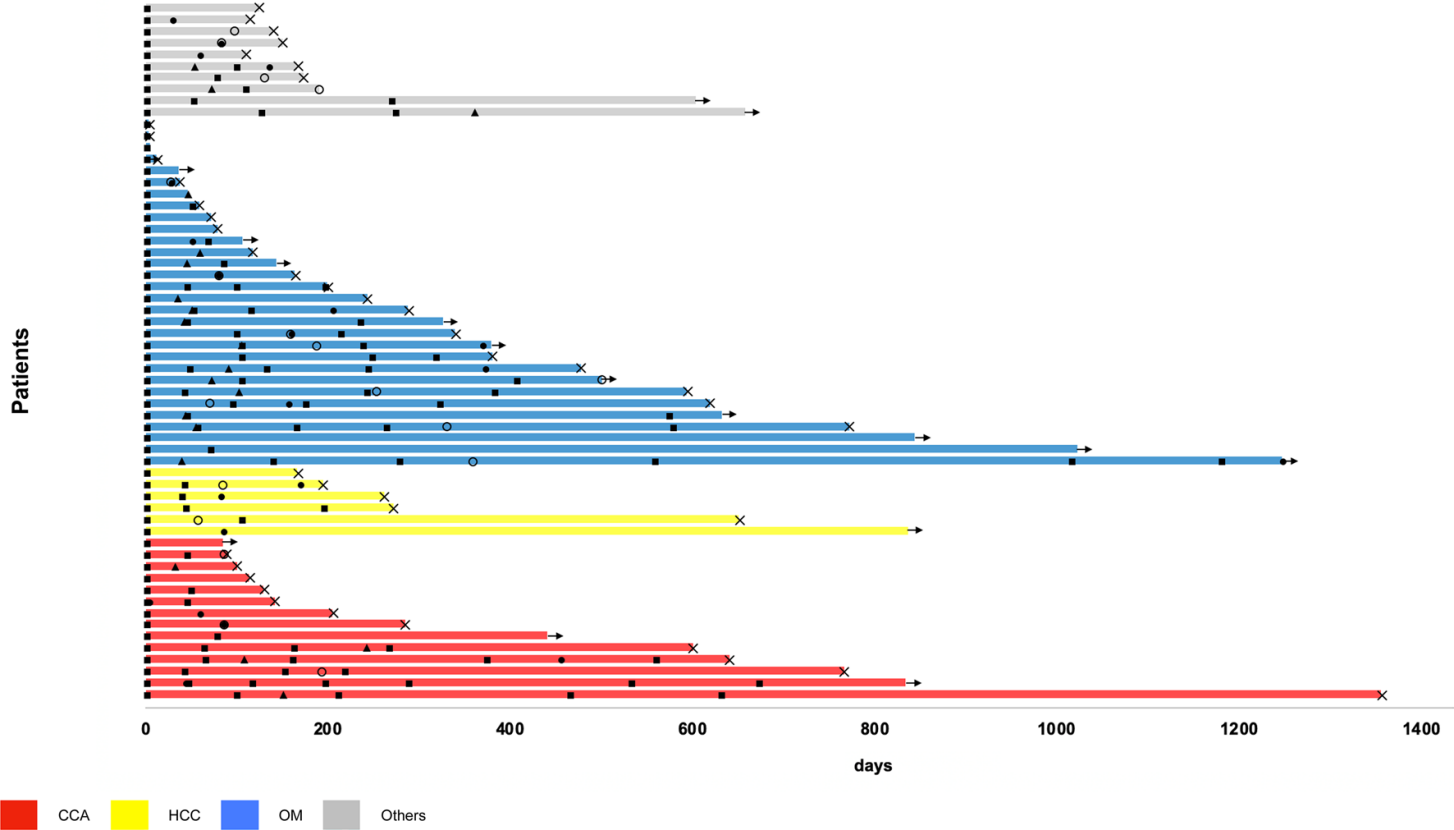
Survival and response assessment. **a** Survival times since first CS-PHP until last follow-up or death including time to response, to first hepatic and extra-hepatic progression and intervals between procedures subdivided by tumor entity

One patient with CCA had the longest survival since first CS-PHP with 3.7 years, followed by three patients with OM and a survival time of 3.4 years, 2.8 years, and 2.3 years, respectively. In total, 16 patients (26.7%) are still on treatment and are being evaluated for further CS-PHS. Among these patients, there are ten patients with OM (33.3%), three patients with CCA (21.4%), one patient with HCC (16.7%), and two patients with other secondary malignancies than OM (20%). Survival times from first CS-PHP until last follow-up or death including time to response, time to hepatic and extra-hepatic progression, and intervals between procedures are represented in Fig. 2.

### Toxicities and complications

**Fig. 3:**
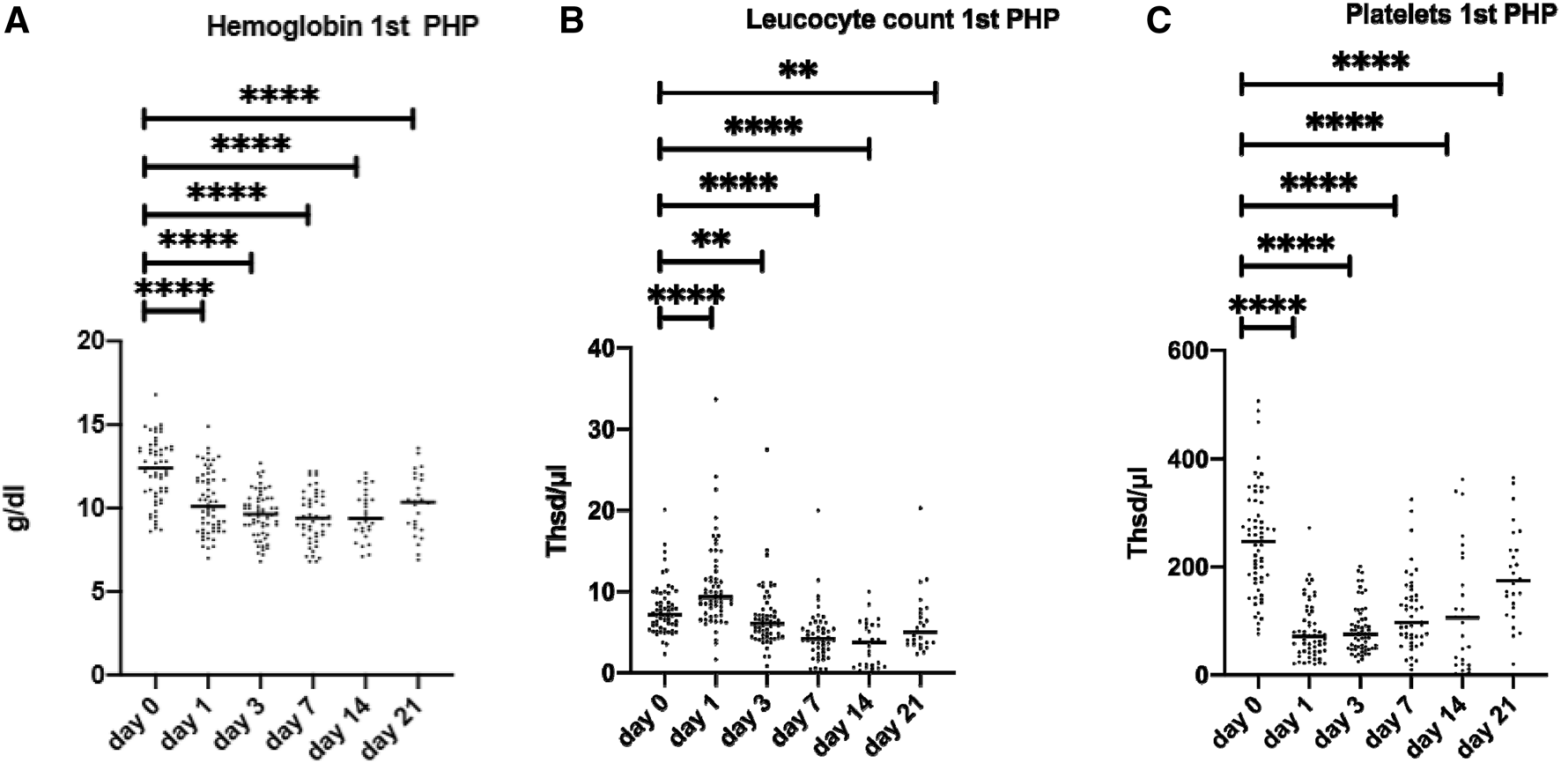
Laboratory values from day 0 of CS-PHP until day 21. Assessment of hematologic function by hemoglobin, leukocyte, and platelet count (**a**–**c**). Pairwise analyses were performed using the Wilcoxon signed-rank test. ****p < 0.0001; ***p < 0.001; **p < 0.01; *p < 0.05

Median time of hospitalization after the first CS-PHP was 7.5 (IQR 6–11) days. The overall rate of grade 3/4 thrombocytopenia, anemia, and leucopenia during treatments with CS-PHPs was 80%, 45%, and 31.6%, respectively. Overall, 30% and 31.7% of the patients needed transfusions of platelet and erythrocyte concentrates, respectively (Table 3). However, myelosuppression was transient and recovered within 21 days after the procedure (Fig. 3a–c). In respect to liver toxicity, there was a significant increase of aminotransferases as markers of hepatic injury. Overall, 48.3% and 26.7% of the patients had a grade 3/4 increase of aspartate transaminase (AST) and of alanine transaminase (ALT), respectively (Table 3). In contrast, liver synthesis function deteriorated less frequently with grade 3/4 hyperbilirubinemia in 15.3% and grade 3/4 hypoalbuminemia in 15.4% of the patients (Table 3). Two patients with OM and a high tumor volume of 32.1% and 72.9% and LDH of 3370 U/L and 3280 U/L died 3 and 12 days after CH-PHP, respectively. In both patients, cause of death was liver failure due to a combination of tumor progression and tumor lysis syndrome.

**Table 3:** Adverse events as assessed by CTCAE v4.03 after first and after overall CS-PHP

	After 1st CS-PHP		Overall	
	*n*	%	*n*	%
Platelet concentrate	12	20.3	18	30.0
Erythrocyte concentrate	11	18.6	19	31.7
Grade 3 thrombopenia	15	25.0	28	46.7
Grade 4 thrombopenia	14	23.3	20	33.3
Grade 3 anemia	19	31.7	26	43.3
Grade 4 anemia	0	0	1	1.7
Grade 3 leucopenia	4	6.7	8	13.3
Grade 4 leucopenia	7	11.7	11	18.3
Grade 3 AST increase	11	18.3	20	33.3
Grade 4 AST increase	7	11.7	9	15.0
Grade 3 ALT increase	4	6.7	12	20.0
Grade 4 ALT increase	2	3.3	4	6.7
Grade 3 hyperbilirubinemia	5	8.3	8	13.6
Grade 4 hyperbilirubinemia	1	1.7	1	1.7
Grade 3 hypoalbuminemia	4	8.7	8	15.4
Grade 4 hypoalbuminemia	0	0	0	0

Major intervention-associated complications were bleeding complications, overhydration, and most seriously cardiovascular events. Ulcerous bleedings occurred in two patients following CS-PHP (3.3%). In one of them, the ulcer was refractory to endoscopic and medical treatments and was treated surgically. Minor bleedings included bleedings from the puncture site, intraocular hemorrhage, and epistaxis. Generalized edema, ascites, and/or pleural effusion due to overhydration and/or hypoalbuminemia occurred in 13 patients (21.7%), and were treated with diuretics and paracentesis.

Cardiovascular complications occurred in three patients (5%). One patient with periampular carcinoma had a total atrioventricular block, which was successfully cardioverted by a precordial thump. One patient with CCA developed a transient hemiparesis 1 day after the fifth procedure. Cerebral MRI revealed a vascular, ischemic insult within the left-sided precentral cortex most likely due to a thromboembolic event. Further cardiovascular and neurologic tests showed no pathological findings. Lysis was not performed in view of the mild symptoms, which improved spontaneously within hours. Another patient with OM presented with increasing disorientation and a hemiparesis 1 day following the first procedure. A cerebral CT scan revealed an occlusion of the left arteria cerebri media. A thrombectomy was immediately performed and the patient received anticoagulation. A subsequent CT scan revealed a demarked ischemic lesion of the right-sided frontal cortex. Further diagnostics were without pathological findings. Unfortunately, the symptoms remained, so that the patient had to be moved to a neurologic rehabilitation clinic for intensified neurologic trainings.

Minor complications included puncture site complications in two patients (3.3%) with a dissection of the common hepatic artery and the development of a femoral pseudoaneurysm in another patient.

## Discussion

CS-PHP is a novel technique, which delivers high doses of chemotherapy directly to liver tumors while limiting systemic toxicity through hemofiltration of the hepatic venous blood. Here, we report the largest real-life cohort of 60 patients with primary and secondary malignancies treated with 141 CS-PHPs in the salvage setting at Hannover Medical School, Germany. This study represents an extension and an update of our previous single-center experience with an enlarged cohort with a longer treatment (October 2014 until January 2019) and follow-up period (median follow-up 27 months) (Kirstein et al. 2017). Here, we support our previous findings that CS-PHP is particularly effective in patients with OM and CCA.

CS-PHP has previously been studied in various cancer types and is currently most widely used in patients with OM based on the results of a phase III trial, showing that treatment with CS-PHP was superior to BAC (Pingpank et al. 2005). First results from a prospective, randomized-controlled trial including the improved, second-generation filter also showed an acceptable safety and toxicity profile in 35 patients treated for liver metastases from OM (Meijer et al. 2019).

In the real-life setting, several retrospective studies have investigated the safety and efficacy of second-generation CS-PHP among various primary and secondary liver tumors—and most frequently in patients with OM (Artzner et al. 2019; Kirstein et al. 2017; Vogl et al. 2014; Marquardt et al. 2019). Patients with OM represent particularly eligible candidates for CS-PHP as these patients exclusively develop hepatic metastases, which are highly sensitive to melphalan (Feldman et al. 2004a; Jovanovic et al. 2013). Moreover, there are no established systemic or alternative locoregional therapies for patients with metastatic OM. Accordingly, the majority of our patients were diagnosed with OM (*n* = 30; 50%). Here, we show that patients with OM had the most favorable course in accordance to the efficacy signals that we have observed within our first CS-PHP evaluations (Kirstein et al. 2017). We also provide evidence that patients with OM and low levels of LDH as a surrogate marker of tumor load represent specifically good candidates for CS-PHP. Patients with OM had in trend a higher ORR, a prolonged mOS, mPFS, and mhPFS compared to the other patients. In patients with OM, ORR was 42.3%, mOS was 12 months, and both mPFS and mhPFS was 6 months. Our data, therefore, confirm phase III study results, which have shown improved control of liver disease in patients with OM treated with CS-PHP with a hepatic objective response rate of 36.4%, an mPFS of 5.4 months, and an mhPFS of 7 months compared to BAC (Hughes et al. 2016). The high crossover of BAC patients to CS-PHP confounded any possible overall survival advantage in this trial. A smaller retrospective study reported even longer survival times in patients with OM reaching up to 27 and 11 months for mOS and mPFS, respectively (Artzner et al. 2019). However, these numbers might be overestimated due to the low number of patients (*n* = 16) and procedures (*n* = 28) in that study.

Patients with biliary cancers represented the second largest subgroup in our cohort (*n* = 14; 23.3%). Patients with biliary tract tumors are frequently diagnosed at advanced stages, when curative surgery is often not possible anymore (Blechacz and Gores 2008). Accordingly, their prognosis is dismal. Systemic therapies represent the standard treatment in the palliative setting. Next to chemotherapies such as gemcitabine/cisplatin and 5-fluoropyrimidines, targeted therapies such as inhibitors of the isocitrate dehydrogenase 1 or fibroblast growth factor receptor 2 (FGFR2) are becoming increasingly relevant (Valle et al. 2010; Lamarca et al. 2019; Abou-Alfa et al. 2019, 2020). Regarding systemic therapies for patients with CCA in the second line, ORRs vary from 8 to 36% for chemotherapy and for FGFR2 inhibition with pemigatinib in selected patients with FGFR2 fusions or rearrangements, respectively (Abou-Alfa et al. 2020; Lamarca et al. 2014). So far, there are no therapeutic standards for patients with unresectable tumors, which are refractory to systemic therapy. There are a few efficacy data on CS-PHP in patients with CCA. Prospective data are available from one, small phase I trial on treatment with the second-generation CS-PHP, which included patients with hepatobiliary cancers among other solid tumors (Pingpank et al. 2005). An early retrospective, multi-center study analyzed CS-PHP treatments in 14 patients, among which one patient with CCA achieved a complete response (Vogl et al. 2014). In our previous single-center experience, patients with biliary tumors were characterized by a long-lasting tumor stabilization with an overall SD rate of 40% (Kirstein et al. 2017). In a further multi-center study, we have exclusively investigated 15 patients with CCA who were treated with CS-PHP in the salvage setting and achieved a considerable ORR of 20% and a median OS of 8 months (Marquardt et al. 2019). In the present study, patients with CCA were again characterized by a long-lasting tumor stabilization and a survival of up to 3.7 years from first CS-PHP and a high ORR of 30.8%. Similarly, to CS-PHP, transarterial radioembolization (TARE) has been evaluated in small cohorts of patients with CCA in the second line and reached a promising 3-months response rate of 35% comparable to our results (Köhler et al. 2019). Treatment with either TARE or CS-PHP has been discussed for each patient within a multidisciplinary tumor board including internal medicine, surgery, nuclear medicine, radiation oncology, pathology, and radiology, whereas TARE was most often not performed due to the lack of clear hypervascularization in pre-interventional imaging and risk for radiation-induced liver disease due to multiple lesions. Head-to-head trials comparing TARE to CS-PHP could be a subject of further research.

There is currently less evidence, whether CS-PHP should be used in patients with HCC or liver metastases from other solid malignancies than OM (Alexander et al. 2005, 2009; Iersel et al. 2018; Feldman et al. 2004b; Grover et al. 2004). Based on the modest benefits, we have observed within our first experiences with CS-PHP, and the fraction of patients with HCC and non-OM metastases among all treated patients decreased from 44.8 to 26.7%. Again, none of the patients with HCC had a radiological response. One patient with HCC had a hepatic response with a decrease of alpha-feto-protein levels, but developed extra-hepatic tumor manifestation at the same time. Considering the novel and effective systemic therapeutics such as atezolizumab and bevacizumab, CS-PHP does not represent a promising treatment strategy in the palliative setting for patients with HCC (Finn et al. 2020). Altogether, CS-PHP in patients with HCC and non-OM metastases should be considered only in particular cases after careful consideration. Accordingly, the number of patients with HCC and non-OM metastases treated with CS-PHP at our institution has constantly decreased over the years (supplemental Fig. 1).

In accordance to other prospective and retrospective studies, hematologic toxicities were significant but manageable and transient in our study (Hughes et al. 2016; Pingpank et al. 2005; Meijer et al. 2019; Artzner et al. 2019; Kirstein et al. 2017; Vogl et al. 2014; Marquardt et al. 2019). Thrombocytopenia was most prevalent next to anemia and leucopenia. In our previous evaluation, the overall rates of grade 3/4 thrombocytopenia, anemia, and leucopenia have been similarly high with 89.7%, 41.3%, and 34.5% compared to the present study with 80%, 45%, and 31.6%, respectively (Kirstein et al. 2017). Based on our initial results, we have implemented the routine use of G-CSF and close monitoring of laboratory values after treatment. However, the rates of grade 3/4 leucopenia have not decreased significantly since then confirming that the initial and immediate decrease of blood cells and also albumin is rather due to the filtration system than to chemotherapy-associated myelosuppression (Moeslein et al. 2014).

Hepatic injury, which manifested as transient increase of aminotransferases, was frequently seen following CS-PHP in accordance to the previous studies (Hughes et al. 2016; Pingpank et al. 2005; Meijer et al. 2019; Artzner et al. 2019; Kirstein et al. 2017; Vogl et al. 2014; Marquardt et al. 2019). Hepatic dysfunction was less often observed with an overall rate of grade 3/4 hyperbilirubinemia of 15.3%. Two patients with OM and a high tumor volume died soon following CS-PHP most probably due to a combination of tumor progression und tumor lysis syndrome. In both patients, CS-PHP had been performed despite the high risk due to the strong therapeutic wish of the patients. In our previous work, we could already show that high tumor volume negatively correlated with survival (Kirstein et al. 2017). These events emphasize again that CS-PHP should be carefully discussed in patients with a high tumor burden. Based on our experiences, we do not recommend CS-PHP at a tumor volume > 50% or LDH of (< 500 U/L) anymore.

Cardiovascular events seem to represent a rare, but very serious complication of CS-PHP. In this study, two patients underwent serious ischemic insults. In one patient, all symptoms have resolved, luckily. More tragically, neurologic symptoms (hemiparesis and disorientation) remained in the other patient, even though an immediate thrombectomy had been performed. Similar to our observations, a few cases of cerebral ischemia and cardiac toxicity including myocardial infarction and arrhythmias have been reported in the phase III melanoma trial (Hughes et al. 2016). Within another retrospective study, one patient suffered from cardiac arrest during treatment, so that treatment was stopped (Artzner et al. 2019). After treatment of an occluded coronary artery, the patient was treated by TARE instead of CS-PHP. As a consequence to the serious vascular events that happened at our institution, we have started to waive the administration of protamine, which had previously been administered to the patients before the removal of large catheters according to the international standards of other referral centers for CS-PHP. These events, furthermore, emphasize that careful patient selection including the identification of potential cardiovascular risk factors, recommendations of cardiologists and neurologists, and close follow-up monitoring is crucial.

The major limitation of our study is the retrospective nature with all its potential confounders. Toxicities and complications may be underestimated. Our results stress the complexity of the procedures, which should be performed with utmost caution by interventional radiologists and anesthesiologists with great expertise. However, this is, to our knowledge, the largest real-life study on CS-PHP in patients with liver tumors as last-line treatment. Our results support the rationale to pursue prospective clinical trials specifically in patients with CCA next to patients with OM.

## Electronic supplementary material

Below is the link to the electronic supplementary material.

Supplementary file1 (PPT 157 kb)

Supplementary file2 (DOCX 22 kb)

## Abbreviations

AFP: Alpha-fetoprotein
ALT: Alanine transaminase
AST: Aspartate transaminase
BAC: Best alternative treatment
CCA: Cholangiocarcinoma
CI: Correlation index
CRC: Colorectal carcinoma
CRP: C-reactive protein
CTCAE: Common Terminology Criteria for Adverse Events
CS-PHP: Chemosaturation percutaneous hepatic perfusion
CT: Computed tomography
ECG: Electrocardiogram
ECOG: Eastern Cooperative Oncology Group
HCC: Hepatocellular carcinoma
mRECIST: Modified response evaluation criteria in solid tumors
OM: Occular melanoma
ORR: Overall response rate
PR: Partial response
PD: Progressive disease
PS: Performance status
RECIST: Response evaluation criteria in solid tumors
SD: Stable disease
TARE: Transarterial radioembolization
ULN: Upper limit of normal
