# Supplementary figures and images for: Chemosaturation with percutaneous hepatic perfusion is effective in patients with ocular melanoma and cholangiocarcinoma
Source: J Cancer Res Clin Oncol. 2020 Jun 20;146(11):3003–12. doi: 10.1007/s00432-020-03289-5 (PMC7519914; doi:10.1007/s00432-020-03289-5)

## Slide 1
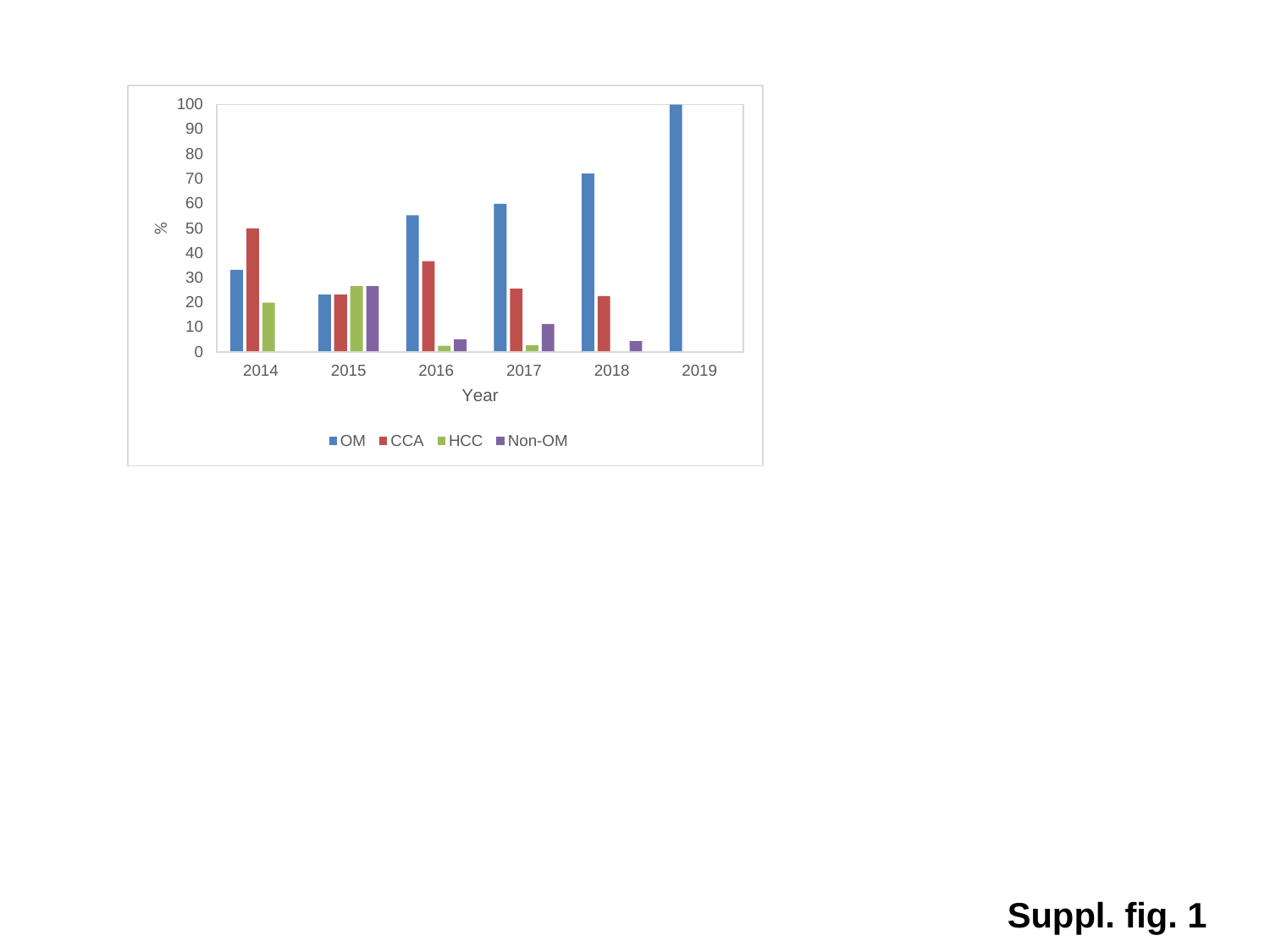

Suppl. fig. 1

Supplement: Supplementary file 1 — Supplementary file1 (PPT 157 kb) [file 432_2020_3289_MOESM1_ESM.ppt]
