# Supplementary material for: Chemosaturation with percutaneous hepatic perfusion is effective in patients with ocular melanoma and cholangiocarcinoma
Source: J Cancer Res Clin Oncol. 2020 Jun 20;146(11):3003–12. doi: 10.1007/s00432-020-03289-5 (PMC7519914; doi:10.1007/s00432-020-03289-5)
Supplement: Supplementary file 2 — Supplementary file2 (DOCX 22 kb) [file 432_2020_3289_MOESM2_ESM.docx]

**Supplemental table 1**

| **Tumour entity** | **Patient number** | **Previous treatments** |
| --- | --- | --- |
| **OM** | 2 | Brachytherapie  Resection of liver metastasis  Liver segment resection |
|  | 6 | Protontherapy  Vitrectomy |
|  | 11 | Enucleation  palliative Chemotherapy (drug unknown) |
|  | 18 | Enucleation |
|  | 19 | Brachytherapy  Micro wave ablation of spleen metastasis  Splenectomy |
|  | 21 | Cyberknife  Ipilimumab  Enucleation  Resection of liver metastasis Radioembolization  Radiatio bone metastasis  Chemotherapy unknown substance |
|  | 24 | Brachytherapy |
|  | \| 25 \| \| --- \| | Brachytherapy |
|  | \| 26 \| \| --- \| | Protontherapy  Trans scleral Thermotherapy  Vitrectomy |
|  | \| 28 \|  \| \| --- \| --- \| | Radiatio  Hemihepatectomy  Liver segment resection |
|  | \| 29 \|  \| \| --- \| --- \| | Avastatin  Radiatio  Enucleation  Fotemustine |
|  | \| 30 \|  \|  \|  \| \| --- \| --- \| --- \| --- \| | Gamma Knife  Enucleation |
|  | \| 33 \|  \| \| --- \| --- \| | Ipilimumab  Pembrolizumab  SIRT |
|  | \| 36 \|  \| \| --- \| --- \| | Brachytherapy  Imatinib  Pembrolizumab |
|  | \| 37 \|  \| \| --- \| --- \| | Enucleation |
|  | \| 38 \|  \|  \|  \| \| --- \| --- \| --- \| --- \| | Brachytherapy  Hemihepatectomy  Nivolumab  Ipilimumab |
|  | \| 42 \|  \|  \|  \| \| --- \| --- \| --- \| --- \| | Gamma Knife  Brachytherapy  Enucleation  Cisplatin / Gemcitabin / Treosulfan |
|  | \| 44 \|  \|  \|  \| \| --- \| --- \| --- \| --- \| | Brachytherapy  Cisplatin / Gemcitabine / Treosulfan |
|  | \| 45 \|  \|  \|  \| \| --- \| --- \| --- \| --- \| | Enucleation |
|  | \| 46 \|  \|  \|  \| \| --- \| --- \| --- \| --- \| | Protontherapy with ablatio  Retinotomie  Avastin |
|  | \| 48 \|  \|  \|  \| \| --- \| --- \| --- \| --- \| | Enucleation  Wedge resection pulmonary upper lobe  Radiatio bone metastasis  Resection of liver segment  Sentinel lymph node biopsy  Radiatio, adjuvant  Tamoxifen |
|  | \| 50 \|  \|  \|  \| \| --- \| --- \| --- \| --- \| | Enucleation  Radiatio bone metastasis  Ipilimumab / Nivolumab |
|  | \| 51 \|  \|  \|  \| \| --- \| --- \| --- \| --- \| | Tantalum Clip Operation  Protontherapy  Vitrectomie  SIRT |
|  | \| 53 \|  \|  \|  \| \| --- \| --- \| --- \| --- \| | Enucleation |
|  | \| 54 \|  \|  \|  \| \| --- \| --- \| --- \| --- \| | Gamma Knife  Vitrectomie  Transretinal endoresection Brachytherapy  Resection of liver metastasis  TACE (Fotemustin / Embocept)  Resection cutaenous metastasis |
|  | \| 55 \|  \|  \|  \| \| --- \| --- \| --- \| --- \| | Liver segment resection |
|  | \| 56 \|  \|  \|  \| \| --- \| --- \| --- \| --- \| | Enucleation |
|  | \| 57 \|  \|  \|  \| \| --- \| --- \| --- \| --- \| | TACE  SIRT  Cisplatin / Dacarbazin / Vindesin |
|  | \| 58 \|  \|  \|  \| \| --- \| --- \| --- \| --- \| | Protontherapy  Vitrectomy  Endoresection |
|  | \| 61 \|  \|  \|  \| \| --- \| --- \| --- \| --- \| | Enucleation  Resection of liver metastasis  Nivolumab / Ipilimumab |
| **CCA** | \| 1 \|  \|  \|  \| \| --- \| --- \| --- \| --- \| | Hemihepatectomy  Gemcitabine / Cisplatin  FUFOX  AIO Scheme |
|  | \| 3 \|  \|  \|  \| \| --- \| --- \| --- \| --- \| | Gemcitabine / Panitumumab  SIRT  Picca Study  Irinotecan / Panitumumab |
|  | \| 9 \|  \|  \|  \| \| --- \| --- \| --- \| --- \| | Hemihepatecotmy  Gemcitabine und Cisplatin  FOLFIRI |
|  | \| 20 \|  \|  \|  \| \| --- \| --- \| --- \| --- \| | Resection of liver metastasis  Gemzar / Cisplatin  Chemoembolisation  Cholezystectomy |
|  | \| 22 \|  \|  \|  \| \| --- \| --- \| --- \| --- \| | Gemzar / Cisplatin  FOLFIRI |
|  | \| 31 \|  \|  \|  \| \| --- \| --- \| --- \| --- \| | Hemihepatectomy  Resection extrahepatic bile duct Gemcitabine |
|  | \| 32 \|  \|  \|  \| \| --- \| --- \| --- \| --- \| | Gemcitabine / Cisplatin  FOLFIRI  Vemurafenib / Cetuximab / Irinotecan  FOLFOX |
|  | \| 34 \|  \|  \|  \| \| --- \| --- \| --- \| --- \| | Resection extrahepatic bile duct  Gemcitabin / Cisplatin |
|  | \| 35 \|  \|  \|  \| \| --- \| --- \| --- \| --- \| | Hemihepatectomy  Cholezystectomy  Gemcitabine / Cisplatin  Photontherapy  Radio frequency ablation  Micro wave ablation  Wedge resection pulmonary upper lobe  Radio frequency ablation |
|  | \| 40 \|  \|  \|  \| \| --- \| --- \| --- \| --- \| | Gemcitabine / Cisplatine |
|  | \| 49 \|  \|  \|  \| \| --- \| --- \| --- \| --- \| | Hemihepatectomy  Gemzar / Cisplatin  FOLFIRI  Gemcitabine  FOLFOX |
|  | \| 52 \|  \|  \|  \| \| --- \| --- \| --- \| --- \| | Naliricc Study: 5-FU/LV  Gemcitabine / Cisplatin  FOLFIRI  FOLFOX  Ivosidenib |
|  | \| 59 \|  \|  \|  \| \| --- \| --- \| --- \| --- \| | Liver segment resection  Gemcitabin / Cisplatin  FOLFIRINOX |
|  | \| 62 \|  \|  \|  \| \| --- \| --- \| --- \| --- \| | Cisplatin / Gemcitabin  Oxallplatin / Capecitabin |
| **Colon** | \| 4 \|  \|  \|  \| \| --- \| --- \| --- \| --- \| | CHARTA Studie (FOLFOX / Avastatin)  FOLFIRI/Avastin  FOLFIRI/Panitumamb  Regorafinib |
| **UC** | \| 17 \|  \|  \|  \| \| --- \| --- \| --- \| --- \| | Zystectomie  Nephrectomie  Wertheim Meigs Operation  Radiatio  Resection of liver metastasis  Carboplatin  Tamoxifen  Hemihepatectomy |
| **HCC** | \| 5 \|  \|  \|  \| \| --- \| --- \| --- \| --- \| | TACE  Radio frequency ablation  Nexavar |
|  | \| 7 \|  \|  \|  \| \| --- \| --- \| --- \| --- \| | Sorafenib  SIRT |
|  | \| 12 \|  \|  \|  \| \| --- \| --- \| --- \| --- \| | TACE  SIRT  Nexavar (Sorafenib Tosylat)  Pembrolizumab  Cabozantinib  Resorce Study Regorafenib |
|  | \| 13 \|  \|  \|  \| \| --- \| --- \| --- \| --- \| | TACE  Sorafenib (Nexavar)  Nivolumab |
|  | \| 15 \|  \|  \|  \| \| --- \| --- \| --- \| --- \| | TACE |
|  | \| 43 \|  \|  \|  \| \| --- \| --- \| --- \| --- \| | Nexavar |
| **MC** | \| 16 \|  \|  \|  \| \| --- \| --- \| --- \| --- \| | Tumorectomy  Radiatio, adjuvant  EC Scheme (Epirubicin, Cyclophosphamid)  Taxol  Navelbine  Calciumfolinat / 5-FU  Ablatio mammae |
| **NET** | 39 | Liver segment resection  Everolimus  Lanreotidtherapy |
|  | \| 47 \|  \|  \|  \| \| --- \| --- \| --- \| --- \| | Pancreas left resection  Splenectomie  Liver section resection  Sandostatin / Afinitor  Sutent  5-FU / Streptozotocin  PRRT  TACE  SIRT  Xeloda / Temodal  Lenvatinib |
| **PC** | \| 14 \|  \|  \|  \| \| --- \| --- \| --- \| --- \| | Whipple Operation  Gemcitabine  FOLFIRINOX |
| **Papille** | \| 8 \|  \|  \|  \| \| --- \| --- \| --- \| --- \| | Whipple Operation  XELOX  Liver section resection  FOLFIRI |
|  | \| 10 \|  \|  \|  \| \| --- \| --- \| --- \| --- \| | Pancreatoduodenectomy  Cholezystectomie  FOLFOX  Gemzar / Cisplatin |
| **PCA** | \| 27 \|  \|  \|  \| \| --- \| --- \| --- \| --- \| | Whipple Operation  Gemcitabin  FOLFOX |
| **CRC** | \| 23 \|  \|  \|  \| \| --- \| --- \| --- \| --- \| | Radio frequency therapy, adjuvant  Resection liver metastasis  Xeloda  FOLFIRI / Avastin (Bevacizumab) |
